# Use of a machine learning framework to predict substance use disorder treatment success

**DOI:** 10.1371/journal.pone.0175383

**Published:** 2017-04-10

**Authors:** Laura Acion, Diana Kelmansky, Mark van der Laan, Ethan Sahker, DeShauna Jones, Stephan Arndt

**Affiliations:** 1 Instituto de Cálculo, Facultad de Ciencias Exactas y Naturales, Universidad de Buenos Aires, CONICET, Buenos Aires, Argentina; 2 Iowa Consortium for Substance Abuse Research and Evaluation, University of Iowa, Iowa City, Iowa, United States of America; 3 Division of Biostatistics, University of California, Berkeley, California, United States of America; 4 Counseling Psychology Program, Department of Psychological and Quantitative Foundations, College of Education, University of Iowa, Iowa City, Iowa, United States of America; 5 Department of Psychiatry, Roy J and Lucille A Carver College of Medicine, University of Iowa, Iowa City, Iowa, United States of America; 6 Department of Biostatistics, College of Public Health, University of Iowa, Iowa City, Iowa, United States of America; Legacy, Schroeder Institute for Tobacco Research and Policy Studies, UNITED STATES

## Abstract

There are several methods for building prediction models. The wealth of currently available modeling techniques usually forces the researcher to judge, a priori, what will likely be the best method. Super learning (SL) is a methodology that facilitates this decision by combining all identified prediction algorithms pertinent for a particular prediction problem. SL generates a final model that is at least as good as any of the other models considered for predicting the outcome. The overarching aim of this work is to introduce SL to analysts and practitioners. This work compares the performance of logistic regression, penalized regression, random forests, deep learning neural networks, and SL to predict successful substance use disorders (SUD) treatment. A nationwide database including 99,013 SUD treatment patients was used. All algorithms were evaluated using the area under the receiver operating characteristic curve (AUC) in a test sample that was not included in the training sample used to fit the prediction models. AUC for the models ranged between 0.793 and 0.820. SL was superior to all but one of the algorithms compared. An explanation of SL steps is provided. SL is the first step in targeted learning, an analytic framework that yields double robust effect estimation and inference with fewer assumptions than the usual parametric methods. Different aspects of SL depending on the context, its function within the targeted learning framework, and the benefits of this methodology in the addiction field are discussed.

## Introduction

There are several methods for building prediction models. Prediction models are often generated using some form of linear or logistic regression—e.g. [1–4]. More recently, other learning algorithms such as random forests (RF) or artificial neural networks (ANN) are being used for prediction in the health sciences—e.g. [5–7]. These new techniques may be able to enhance prediction, thus improving the chances of matching patients to the most effective treatments.

The wealth of currently available modeling techniques usually forces the researcher to judge, a priori, what will likely be the best prediction method. Super learning (SL) [8] is a methodology that facilitates this decision by combining all identified prediction algorithms pertinent for a particular prediction problem. SL generates a final model that is at least as good as any of the other models considered for predicting the outcome. This property of SL is both theoretically [8] and empirically supported [9]. The goal of this paper is to introduce various prediction methods, some of which are novel to the field of substance use disorders (SUD) treatment.

Accounting for individual patient characteristics to maximize positive outcomes is at the heart of precision medicine. SUD treatment is one of the many areas that can greatly benefit from optimizing patients’ pathways to the best possible treatment outcome. For example, United States estimates indicate only 19.8% of cases in need of alcohol use disorders treatment were ever treated [10]. Identifying key predictors of successful treatment can serve to discover disparities, strengths and weaknesses in service delivery, eventually increasing treatment success and reducing unmet treatment needs [11–13].

The first step in identifying key characteristics for matching patients to the most effective treatments is to predict who will succeed at a given treatment. The importance of this topic is reflected by the rich literature aiming to identify patients’ characteristics increasing the efficacy of SUD treatment (e.g. [4, 14–16]). The methodological focus of this paper leaves a substantive literature review regarding SUD treatment success out of its scope.

Some of the literature predicting successful SUD treatment takes advantage of large publicly available datasets such as the Substance Abuse and Mental Health Services Administration Treatment Episode Data Set—Discharges (TEDS-D) [17]. The main advantage of datasets such as TEDS-D is the large number of records available including important patient characteristics and treatment features. Due to the computer power required when large data sets with numerous predictors are used, the advantage of working with large datasets or big data used to be also the greatest disadvantage for applying sophisticated prediction algorithms such as SL or other sophisticated machine learning methods. Computer power is currently less of a problem thanks to technological advances and an active open source software developer community. For instance, the open source community has optimized SL and other machine learning algorithms for the analysis of big data using open source software [18].

Most of the methods featured in this paper were not used to analyze TEDS-D before. The aim is to evaluate what method works best at predicting successful treatment using a real life large database. Rather than a dataset tailored to showcase the methods properties, we use a dataset commonly used in the SUD field. We hypothesized that SL will generate the best prediction model as measured by the area under the receiver operating characteristic curve (AUC) evaluated in a test sample not included in the training sample used to fit all prediction models.

The use of SL as a prediction tool of success in SUD treatment may contribute to the literature by bettering the ability to identify treatment outcome disparities that, when addressed, may lead to improve patients’ treatment outcomes.

## Materials and methods

### Data

To illustrate different prediction analytic approaches, we focused in SUD outpatient treatment for Hispanics during adulthood using publicly available TEDS-D 2006–2011 data [17]. TEDS-D is an excellent example of a large administrative dataset that may be of interest to addiction researchers in real life. This dataset allows us to illustrate the use of the methodologies introduced in this work within a realistic setting.

#### Outcome

Treatment completed was considered a successful treatment discharge status, all other treatment discharge reasons (e.g., left against professional advice, incarcerated, other) were considered as indicators of non-successful treatment. Treatment completion is a standard process outcome measure because it predicts longer-term outcomes such as less future criminal involvement, fewer readmissions, employment and income one year after treatment [19–22].

#### Predictors

Twenty eight predictors recorded by TEDS-D were included in the analysis. Predictors include 10 patient characteristics (i.e., age, gender, race–e.g., White, Black—, ethnicity–indicating patient’s specific Hispanic origin, marital status, education, employment status, pregnant at time of admission, veteran status, and living arrangement), 3 treatment characteristics (i.e., intensity, medication-assisted opioid therapy, and length of stay), principal source of referral, summary of type of problematic substance (with categories “alcohol only”, “other drugs only”, or “alcohol and drugs”), and mental health problem. TEDS-D records thorough information about substances of misuse. This includes the following 12 predictors: primary, secondary, and tertiary substance problem, usual route of administration, frequency of use, and age at first use. The substances include: alcohol, cocaine/crack, marijuana/hashish, prescription opiates/synthetics, and methamphetamine use. Several other drug use categories were collapsed for analysis because of low percentages.

#### Inclusion criteria

TEDS-D includes all admissions/discharges rather than individuals. Consequently, only records that indicate the individual had no prior SUD treatment were included in the analyses. Part of our previous work focuses on racial and ethnic minorities [11, 12, 23, 24]. It is known that racial and ethnic minorities vary in their treatment access and success levels [25–27]. Thus, we restricted our analysis only to cases indicating a Hispanic/Latino ethnicity, 18 years old or older, and treatment in outpatient service settings. We focused on outpatient service settings because criteria for treatment completion/success (i.e., the outcome of interest) and duration for other types of services (e.g., 24-hour inpatient, detoxification-only) and outpatient services are often very different. This is a sample arbitrarily chosen to exemplify the different analytical approaches. The choice is based on our previous knowledge in this field and not on the results obtained after using the methods described in the following paragraphs.

#### Exclusion criteria

Records with missing data in any of the predictors, the outcome, or characteristics used for determining inclusion in the study were excluded from the analysis. Since not all states collect the same information for their patients, from a total of 9,829,536 records, 4,385,825 (45%) had all required data complete.

These inclusion and exclusion criteria did not affect the performance of SL when compared to the rest of the analytical strategies used. A total of 99,013 records representing unique individuals were selected according to the inclusion and exclusion criteria and were used in the analysis. This sample was separated in a training set with 80% of the sample (n = 79,210) and a test set with the remaining 20% (n = 19,802).

Because these data represent public information and there is no subject identification, the University of Iowa Human Subjects Office Institutional Review Board exempted this study from review.

### Statistical analysis

The goal of the analysis was to compare results of classical analytical strategies that are commonly used to address prediction of treatment success (e.g., logistic regression) alongside results of newer methods for prediction (e.g., ANN). We also compared SL results. SL is an ensemble machine learning methodology that encompasses all other methodologies in its library and has demonstrated (both theoretical and empirical) superiority for prediction [8, 9].

Formally, the analysis is as follows. Let *W = {W*_*1*_, *…*, *W*_*k*_*}* denote the predictors of interest and *Y* represent the binary outcome treatment success (Yes/No). Let *O = (W*, *Y)* be a random variable such that *O~P*_*0*_ (i.e., the true probability distribution of *O* is *P*_*0*_). Since only records that indicate the individual had no prior treatment in a drug or alcohol program were included in the analyses, we assumed that the individuals observed can be represented as independent and identically distributed observations of the random variable *O*. For each individual *i*, *Y*_*i*_ and *W*_*i*_ are observed. We sought to estimate ${\bar{Q}}_{0}=P_{0}(Y=Yes|W)$—i.e., the probability of succeeding in treatment given the predictors of interest. ${\bar{Q}}_{0}$ is unknown. Hence, we aimed to find the best estimator of ${\bar{Q}}_{0}$. This was achieved by maximizing the AUC. We did this using logistic regression, 3 types of penalized regression, RF, ANN, and the corresponding SL that includes these algorithms in its library. SL also maximized the AUC.

**Fig 1:**
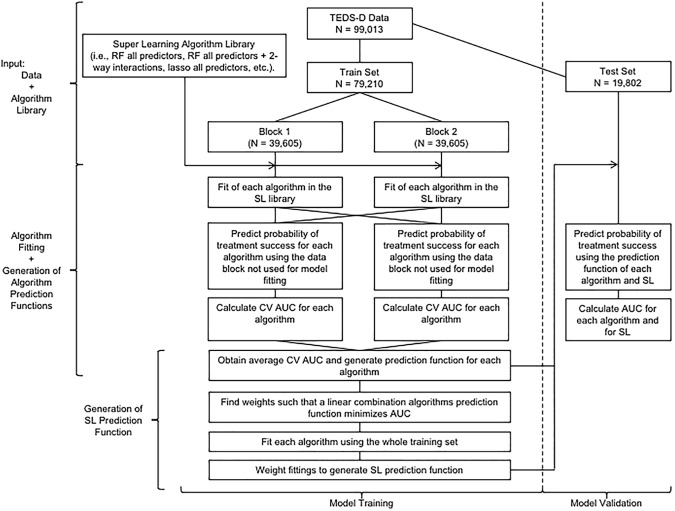
Analytic work flow.

We used 2 mechanisms to avoid overfitting. The analysis used 2-fold cross-validation (CV) using 80% of the data set (n = 79,210) and the final model was validated using 19,802 randomly selected individuals that were only included in the test set (Fig 1). All analytic approaches explored were compared using the AUC in the test set. The best analytic strategy for predicting treatment success was identified as the model maximizing AUC. The AUC is a convenient measure of prediction success that can be interpreted as the probability that any of the algorithms ranks a randomly chosen successful patient higher than a randomly chosen unsuccessful patient [28]. AUC ranges from 0 to 1, AUC = 1 means perfect prediction and AUC = 0.5 suggests chance levels of prediction.

We used at least 2 different configurations for each analytic strategy compared. The first approach included all 28 predictors available. Since it can be useful to reduce the number of predictors considered and simplify the final prediction formula, we also selected a subset of the 10 predictors with highest variable importance in a RF model. In decreasing order of importance, these predictors were: length of stay, age, principal source of referral, primary problematic substance, its age of first use, and its frequency of use, employment level, SUD type (i.e., only alcohol, only drugs, or both alcohol and drugs), education level, and patient’s specific Hispanic origin (e.g., Puerto Rican, Mexican, Cuban).

For each set of predictors, the following algorithms were compared: logistic regression, least absolute shrinkage and selection operator (lasso), ridge, and elastic net penalized regressions [29], RF [30], ANN [29, 31], and SL [8]. Since none of the aforementioned regression models considered interaction effects in the predictive model, an additional model including terms for all predictors and selected 2-way interactions was also added to the SL library for each type of regression. Two-way interactions were initially screened using all possible logistic regression models of 2 predictors at a time and their 2-way interaction. All interactions with p-values<0.0001 were included along with all predictors in the regression models of the SL library. We compared 17 algorithms/algorithm configurations.

There is a multitude of other analytic strategies that could be chosen [29]; however, to illustrate SL use for predicting treatment success when compared to other methods, we consider the chosen algorithms are adequate. In this context, where the data generating model is unknown, SL will be superior to the rest of the methodologies, regardless of the set of algorithms initially chosen. Some characteristics of the chosen algorithms are presented subsequently.

#### Logistic regression

Logistic parametric regression is the most commonly used algorithm for prediction in the SUD treatment outcomes field. Logistic regression is easily implemented and interpreted. However, logistic regression assumptions are strong and, since the true data generating model is unknown, these assumptions are usually violated. For example, including many predictors, their interactions, and/or other higher order terms in a logistic regression model does not guarantee that it is the best model, due, for example, to collinearity between the predictors included (which increases variability) or model misspecification (which introduces bias).

#### Penalized regression

Penalized regression (such as lasso, ridge regression, or elastic nets) offers an alternative to parametric regression models. The 3 types of penalized regression applied in this work vary in their variance/bias tradeoffs depending on the characteristics of the predictor set. For example, lasso will select only one term from a set of correlated predictors. This may not be appropriate. In fact, when the number of predictors is small compared to the number of independent observations, ridge regression outperforms lasso when variables are highly correlated. Additionally, if the true data generating model has only a few predictors but the candidate models have a large number of predictors; lasso may eliminate predictors that were not in the data generating model. On the other hand, ridge regression will keep all terms in the final model. The elastic net penalized regression provides some balance between lasso and the ridge regressions.

In the example presented here, there are 28 categorical predictors that correspond to 135 terms when the model is parametrized using dummy variables. The screening step for the most relevant 2-way interactions of these 135 terms, preselected 257 2-way interaction terms. Thus, the regression models including all predictors and selected interactions had 393 terms including the intercept. While logistic regression estimated 393 parameters for this model, lasso estimated parameters only for terms uncorrelated with each other and zeroed-out the rest; ridge regression kept all 393 but down weighted each term. In this way, penalized regression allows for tuning large models adapting them to the information provided by the data.

#### Random forests

RF is a recursive partitioning method popular in many fields with high-dimensional data (e.g., genomics). RF can evaluate a number of predictor variables even in the presence of complex interactions, including those that are not possible to model using regression. RF is an ensemble of classification and regression trees constructed on bootstrap samples. Unlike individual trees, RF is more protective against overfitting.

#### Artificial neural networks

An ANN uses interconnected nodes within various layers to explain an outcome given a set of predictors. The relationships between the nodes are defined by weights calculated using a given rule. The initial weights are preassigned by the analyst. The ANN algorithm iterates adjusting the weights. At the end of each iteration, the performance at outcome prediction is evaluated. ANNs efficiently generate non-linear classification rules but can be prone to overfitting. More recently, some types of ANN are referred to as deep learning [31]. Deep learning allows modeling multiple levels of non-linearity in the data and is scalable to large datasets and big data in general. We used deep learning ANN with hidden layer sizes of 200.

#### Super learning

SL is a generalization of the stacking algorithm [32], an ensemble machine learning method that takes a weighted average of all other algorithms considered for prediction and produces a single prediction function (PF) with optimal tradeoff between variance and bias. SL is very flexible for learning from the data as it combines the strengths of all methodologies considered (including different configurations of the same algorithm) while minimizing modelling flaws. Another advantage of SL is that it eliminates the need to select a priori a single or a few methodologies for the analysis. SL allows analyzing the data using simultaneously all the methodologies the researcher considers suitable.

Fig 1 depicts the work flow used to analyze the data and details all the steps necessary for running SL. The input of the analysis consists of the training and test data sets together with the algorithmic library. Since we used 2-fold CV to obtain each algorithm PF, as well as, the SL PF, the training set was initially partitioned in 2 blocks. Each algorithm in the library was fitted using each block independently. We used the data block excluded from the model fitting to calculate the CV AUC for each algorithm in the library. We averaged both CV AUCs, resulting in a single training set CV AUC for each algorithm. Up to this step, model fitting follows a regular 2-fold CV modelling path. The PF of discrete SL, a simpler version of SL, is the PF of the algorithm with the minimum CV AUC.

However, SL performs better when a weighted combination of the algorithms’ PFs is used. Thus, the next step for obtaining SL PF is to calculate a weight for each algorithm PF. This is done regressing *Y* on the values of *Y* predicted by each algorithm in the library. Next, each algorithm is fitted using the whole training set and the SL PF is obtained by applying the estimated weights to the algorithm predictions for each observation.

It can be demonstrated that using 2-fold CV, the procedure can end here and the AUCs of fitting each algorithm and SL to the whole training dataset would suffice for SL to outperform the rest of the algorithms without overfitting. However, we included an additional validation step: all the PFs obtained with the training set, where used to predict SUD treatment success in the test data set. We calculated AUCs compared to evaluate all models adjusted using the test dataset.

A thorough description of all the algorithms used in this work is out of the scope of this manuscript. The interested reader will find further details about SL in van der Laan and Rose [33] and about the rest of the aforementioned methodologies in Friedman et al [29] and Bengio [31].

Models were fitted using the open source R programming language [34] and the H2O R interface version 3.8.2.2 [35] that optimizes all the analytical methods used for large datasets. The h2oEnsemble package version 0.1.8 [36] was used to fit the SL model. We set all tuning parameters for each algorithm in the SL library (e.g., the ANN implementation used in this manuscript has over 20 parameters) to their default values. The analysis took about 2.5 hours to run in a Windows 7 Professional desktop computer with 8-core i7-3770 3.40 Ghz CPU and 8 Gb RAM. Most of the analytic time was used to fit the four regression models including 2-way interaction terms. The other 13 algorithms, including SL, required only about 6 minutes of the 2.5 hours. AUC confidence intervals and variances were estimated using the Delong and colleagues methodology [37] as implemented in the R package pROC [38]. Briefly, Delong et al [37] used the equality between AUC and the Mann-Whitney U statistic and asymptotic normality to analytically derive variance and confidence interval estimators for AUC.

## Results

Treatment success rate and characteristics for the patients included in the analyses are shown in Table 1. For brevity, only gender and the top 10 most important predictors as identified by RF were included in the table.

**Table 1:** Sample characteristics (N = 99,013).

		Total N (%)
**SUD Treatment Success**		
	Yes	44,748 (45.2%)
	No	54,265 (54.8%)
**Gender**		
	Male	77,123 (77.9%)
	Female	21,890 (22.1%)
**Ethnicity**		
	Puerto Rican	31,047 (31.4%)
	Mexican	25,190 (25.4%)
	Cuban	2,683 (2.7%)
	Other/Unspecified	40,093 (40.5%)
**Age (years)**		
	18–20	11,479 (11.6%)
	21–24	16,886 (17.1%)
	25–29	19,625 (19.8%)
	30–34	15,121 (15.3%)
	35–39	11,725 (11.8%)
	40–44	9,436 (9.5%)
	45–49	6,988 (7.1%)
	50–54	4,172 (4.2%)
	55+	3,581 (3.6%)
**Education (years)**		
	<9	17,170 (17.3%)
	9–11	31,507 (31.8%)
	12	34,329 (34.7%)
	13–15	13,062 (13.2%)
	16+	2,945 (3.0%)
**Employment**		
	Full Time	34,586 (34.9%)
	Part Time	10,392 (10.5%)
	Unemployed	29,635 (29.9%)
	Not in Labor Force	24,400 (24.6%)
**Primary Substance**		
	Alcohol	50,782 (51.3%)
	Marijuana	26,269 (26.5%)
	Cocaine	8,554 (8.6%)
	Non-Prescription Opiates	7,791 (7.9%)
	Methamphetamine	2,312 (2.3%)
	Prescription Opiates and Synthetics	2,145 (2.2%)
	Hallucinogens	293 (0.3%)
	Other Sedatives	320 (0.3%)
	Other Stimulants	234 (0.2%)
	Other	313 (0.3%)
**Frequency of Primary Substance Use**		
	Not in the past month	41,529 (41.9%)
	1–3 times past month	20,766 (21.0%)
	1–2 times past week	11,770 (11.9%)
	3–6 times past week	8,272 (8.4%)
	Daily	16,676 (16.8%)
**Age of First Primary Substance Use (years)**		
	<10	4,825 (4.9%)
	12–14	17,577 (17.8%)
	15–17	31,306 (31.6%)
	18–20	22,949 (23.2%)
	21–24	10,895 (11.0%)
	25–29	5,768 (5.8%)
	30–34	2,535 (2.6%)
	35–39	1,573 (1.6%)
	40–44	816 (0.8%)
	45–49	435 (0.4%)
	50–54	211 (0.2%)
	55+	123 (0.1%)
**Substance Abuse Type**		
	Alcohol Only	34,827 (35.2%)
	Other Drugs Only	29,887 (30.2%)
	Alcohol and Drugs	34,299 (34.6%)
**Source of Referral**		
	Self	16,910 (17.1%)
	Alcohol/Drug Abuse Care Provider	3,655 (3.7%)
	Other Health Care Provider	4,013 (4.1%)
	School	341 (0.3%)
	Employer	1,350 (1.4%)
	Other Community Referral	15,503 (15.7%)
	Criminal Justice Referral	57,241 (57.8%)
**Length of Stay (days)**		
	1–30	19,942 (20.1%)
	31–60	15,296 (15.4%)
	61–90	12,476 (12.6%)
	91–120	12,397 (12.5%)
	121 or longer	38,902 (39.3%)

Table 2 shows the AUC in the test set (N = 19,802) for the 17 algorithms/algorithm configurations compared. All AUC are between 0.793 and 0.820. This indicates that, for this set of models, the probability that any of the algorithms will rank a randomly chosen successful patient higher than a randomly chosen unsuccessful patient is between 0.793 and 0.820. This is usually considered a good performance for prediction models.

**Table 2:** AUC in the test set (N = 19,802) for each algorithm and algorithm parametrization used.

Model	AUC	${\hat{\sigma }}^{2}$
Super Learning	0.820	0.165
Random Forests All Predictors	0.816	0.173
Lasso All Predictors + 2-Way Interactions	0.805	0.185
Lasso All Predictors	0.805	0.185
Elastic Net All Predictors	0.805	0.185
Logistic Regression All Predictors	0.805	0.185
Ridge Regression All Predictors	0.805	0.185
ANN Top 10 Predictors	0.805	0.185
Elastic Net All Predictors + 2-Way Interactions	0.804	0.186
ANN All Predictors	0.803	0.186
Lasso Top 10 Predictors	0.801	0.189
Elastic Net Top 10 Predictors	0.801	0.189
Ridge Regression Top 10 Predictors	0.801	0.189
Logistic Regression Top 10 Predictors	0.801	0.189
Random Forests Top 10 Predictors	0.797	0.191
Ridge Regr. All Predictors + 2-Way Interactions	0.793	0.197
Logistic Regr. All Predictors + 2-Way Interactions	0.793	0.197

**Fig 2:**
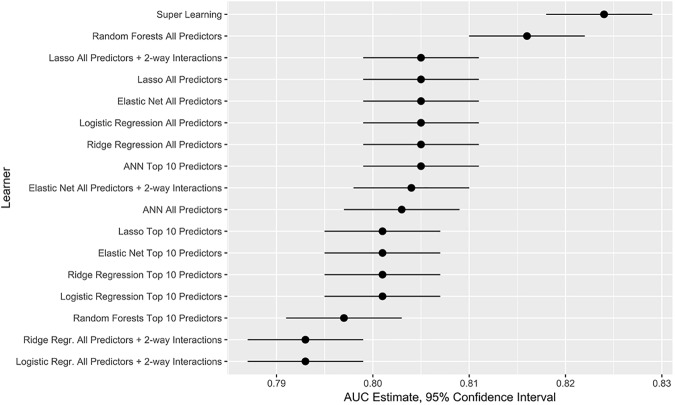
95% confidence intervals for AUC of each model compared.

As hypothesized, SL shows the largest AUC. SL performance is very closely followed by RF including all predictors. For this particular example, the algorithm with the worst performance is logistic regression including all predictors and selected 2-way interactions. The relative improvement in AUC of SL is 3.3% when compared to the worst prediction method. Also, the AUC for SL has the smallest estimated variance. The rest of the models considered have estimated AUC variances up to 19% higher than SL. As revealed by Fig 2, SL AUC is higher than AUC for all other models, with the exception of the RF model including all predictors.

Also, in most cases, the models including all predictors performed slightly better (but not statistically so) than the models including only the top 10 predictors selected in the initial screening step to simplify the prediction model. All parametric regression models both penalized and non-penalized approaches performed almost identically with respect to AUC for models not including interactions. This behavior could have been anticipated since a model with only 28 predictors can be considered too small for a data set this size. However, for models including all predictors and selected 2-way interactions, lasso outperformed the other 3 regression models. AUC for lasso was higher than for ridge and logistic regressions.

## Discussion

This work compared 17 models for predicting successful completion of SUD treatment. Both traditional and newer analytic strategies were equated in a nationwide large dataset. Particularly, super learning, an ensemble machine learning algorithm, was introduced for the first time to the study of SUD treatment success using large datasets. As expected, SL showed the best predictive performance.

In this particular example, SL superiority was meager when compared to more traditional models such as logistic regression. This result was not at all evident before analyzing these data. We understand this may dampen the interest in this work. However, we think that the finding that there are no major differences in the solutions of all methods is important in itself and worth discussing. Unlike what happens with other predictive models, the lack of substantive difference between SL and the rest of the models used means that: a) any of these methods could be used for these data and b) there are no major problems in the assumptions of the different models used. These findings are never evident before or after analyzing a dataset when analysis is performed using a traditional analytical approach. Sensitivity analysis is one approach used to address this limitation of traditional methods. In this sense, SL serves as a tool to streamlining and improving sensitivity analysis for prediction. SL does not require that a model is chosen a priori, at the end of the analysis results in the best model, and, if there are no substantial differences with the rest of the models, SL provides a solid justification for the use of any of the models. SL is a valuable, but underutilized, tool for obtaining robust prediction results as required, for example, by the National Institutes of Health [39].

In addition, even small improvements in prediction can have a high impact depending on each particular problem. In this case, a small prediction improvement could significantly impact patients’ outcomes and/or treatment costs. In other cases, small prediction improvements could save lives. Since SL is theoretically proven to perform as well or better than the best model included in its library, the results presented here are relevant to illustrate the use of SL in the SUD treatment outcome prediction field.

In other applications, SL has shown 44% and 12% increase in performance when compared to ANN and RF, respectively [40]. Also, SL has shown as good or significantly better predictive performance than linear regression and other methods when used in 13 real datasets—see Fig 3.4 in [40]. In fact, stacking algorithms similar to SL are usually among the type of algorithms that win prediction contests such as the Heritage Provider Network Health Prize [41]. The Heritage Provider Network Health Prize was a competition to develop a predictive algorithm helping identify patients most likely to be admitted to healthcare providers.

The results of this work should be interpreted as an illustration of the presented statistical methods in a realistic setting. The practical conclusions should be seen in light of the data limitations. Analysis was restricted to Hispanics and very few features describing the type of treatment and how treatment was administered were included. There may be considerable reservation with using treatment completion as an indicator of treatment success. While post-treatment follow-up outcome measures of abstinence or reduced use would be best, such nation-wide data collection efforts were out of the scope of this project. While these are important limitations to inform treatment success prediction, these issues do not affect the main goal of this work.

Traditional analytical approaches for prediction may not only consider the prediction performance of a model, but also its ecological validity for a given research area. That is one of the reasons logistic regression could be preferred over SL for predicting SUD treatment success. While the model proposed by SL is designed to excel at prediction, it is not meant to be interpreted or to be used for effect estimation. If the true or close to true data generating model is included in the SL library, SL will likely have a low gain in predictive performance and its lack of interpretability could discourage its use. However, most of the time, researchers are uncertain about the model that generated the data. When ecological validity is important in a prediction context, SL could be used as a simpler model validator if an interpretable model in its library is close to SL in predictive performance. In the example presented here, SL has significantly better predictive performance than logistic regression; thus, it would be difficult to defend any of the logistic regression models used in this application in lieu of SL. On the other hand, these data show that, if the ecologically validity of RF models could be easily assessed, the use of the RF model with all the predictors could be used instead of the SL model.

When the goal of the research is to interpret the effect of different predictors in the outcome, SL can be used in the context of the targeted learning framework [33]. SL is the first stage for obtaining doubly-robust effect estimates using targeted maximum loss-based estimation (TMLE) [42]. TMLE is an estimation technique that allows for double robust estimation of effects with fewer untestable assumptions than the usual parametric methods used for estimation. TMLE can be applied either in a causality framework or in a merely associative framework (additional details about TMLE is out of the scope of this work, we recommend [33] for further information). Using the prediction model suggested by SL and TMLE, one could answer questions such as “What is the treatment success rate difference between Hispanics with comorbid psychiatric disorders and those without comorbid disorders?” and “Is this difference different from zero?” In contrast, statistical inference is not possible in machine learning methodologies such as RF or deep learning. This important limitation of machine learning algorithms is overcome by targeted learning, the first analytical framework that provides estimates and hypothesis testing while using machine learning [43]. Future directions of this work include determining the benefits of applying targeted learning for different effect estimations and inference in the addiction field.
